# Identification of potential crucial cuproptosis-related genes in myocardial ischemia-reperfusion injury through the bioinformatic analysis

**DOI:** 10.1016/j.clinsp.2024.100410

**Published:** 2024-06-19

**Authors:** Rong Huang, Rongfeng Xu, Rui Zhang, Wenjie Zuo, Zhenjun Ji, Zaixiao Tao, Yongjun Li, Genshan Ma

**Affiliations:** a Department of Cardiology, Zhongda Hospital, School of Medicine, Southeast University, Nanjing, China; b Department of Cardiology, Affiliated Hospital of Nantong University, Nantong, China

**Keywords:** Cuproptosis, Myocardial ischemia-reperfusion injury, Dlat, Pdhb, Pdhα1

## Abstract

**Background:**

Cuproptosis is known to regulate diverse physiological functions in many diseases, but its role in regulating Myocardial Ischemia-Reperfusion Injury (MI/RI) remains unclear.

**Methods:**

For this purpose, the MI/RI microarray datasets GSE61592 were downloaded from the Gene Expression Omnibus (GEO) database, and the Differently Expressed Genes (DEGs) in MI/RI were identified using R software. Moreover, the MI/RI mice model was established to confirm further the diagnostic value of Pyruvate Dehydrogenase B (Pdhb), Dihydrolipoamide S-acetyltransferase (Dlat), and Pyruvate dehydrogenase E1 subunit alpha 1 (Pdhα1).

**Results:**

The analysis of microarray datasets GSE61592 revealed that 798 genes were upregulated and 768 were downregulated in the myocardial tissue of the ischemia-reperfusion injury mice. Furthermore, Dlat, Pdhb, Pdhα1, and cuproptosis-related genes belonged to the downregulated genes. The receiver operating characteristics curve analysis results indicated that the Dlat, Pdhb, and Pdhα1 levels were downregulated in MI/RI and were found to be potential biomarkers for MI/RI diagnosis and prognosis. Similarly, analysis of Dlat, Pdhb, and Pdhα1 levels in the MI/RI mice revealed Pdhb being the key diagnostic marker.

**Conclusions:**

This study demonstrated the prognostic value of cuproptosis-related genes (Dlat, Pdhb, and Pdhα1), especially Pdhb, MI/RI, providing new insight into the MI/RI treatment.

## Highlights

• Cuproptosis-related genes are involved in acute myocardial infarction progression.
• Dlat, Pdhb, and Pdhα1 levels were downregulated in acute myocardial infarction.
• Dlat, Pdhb, and Pdhα1 may be diagnostic markers in acute myocardial infarction.
• Pdhb showed the best diagnostic value for acute myocardial infarction.

## Introduction

Acute Myocardial Infarction (AMI) is an acute and critical manifestation of coronary atherosclerotic heart disease, with a high incidence rate and mortality.^1^ In recent years, the number of AMI cases has increased dramatically with significantly changed lifestyles, becoming the leading cause of hospitalization and death in China.^2^ Therefore, early and rapid restoration of coronary blood flow is envisaged to reduce the area of myocardial infarction. The AMI mortality rate has declined with the development of reperfusion strategies such as thrombolysis and percutaneous coronary intervention.^3^^,^^4^ However, evidence showed that reperfusion can cause secondary damage to the myocardium, accounting for 50% of the final myocardial infarction area,^5^, ^6^, ^7^ a phenomenon known as Myocardial Ischemia-Reperfusion Injury (MI/RI), which has become the most common clinical problem of AMI after interventional or thrombolytic therapy.^8^ MI/RI can manifest in multiple ways, which can further aggravate the damage to cardiac structure and function and ultimately cause a variety of adverse cardiovascular outcomes.^9^ The occurrence and development of MI/RI involve various pathological processes, such as inflammatory response, oxidative stress, calcium overload, and immune response.^10^^,^^11^ Therefore, actively exploring the molecular mechanism of MI/RI and mining key genes are essential for preventing and treating MI/RI.

In recent years, bioinformatics technology based on gene expression profiles has been widely used to analyze disease-related Differentially Expressed Genes (DEGs), explore key genes, and screen biomarkers related to disease diagnosis, treatment, and prognosis.^12^^,^^13^ DEGs may provide valuable information for studying the development and prevention of MI/RI pathological processes.^14^ Tsvetkov et al.^15^ proposed a copper-dependent novel cell death mechanism called cuproptosis, which differs from other known mechanisms like apoptosis, pyroptosis, and necroptosis. Similar to ferroptosis, Cu^2+^ induces the aggregation of lipoacylated proteins and the instability of iron-sulfur cluster proteins by directly combining with the lipoacylated part of the tricarboxylic acid cycle, leading to proteotoxic stress, thereby inducing cell death independent of the apoptotic pathway.^16^^,^^17^ However, whether cuproptosis occurs in the pathological process of MI/RI is still unclear.

Therefore, this study aimed to analyze expressions of cuprotosis-related genes in MI/RI to demonstrate whether cuprotosis-related genes can be used as diagnostic markers of MI/RI via mouse GSE61592 datasets from the GEO database and establish a MI/RI mice model to validate the findings further. Moreover, potential intervention targets and molecular mechanisms in the pathological process of MI/RI were also explored to provide novel treatment strategies for MI/RI.

## Materials and method

### Blood samples collection

A total of 20 MI/R patients undergoing treatment in the hospital were recruited from June 1, 2022, to June 1, 2023. In addition, 20 healthy individuals who underwent physical examinations at the studied hospital during the same period were recruited as a control group. The serum samples were collected and stored at -80°C for the next experiments. This study was approved by the ethics committee of the hospital (2020ZDSYLL082-P01) and received informed consent from all participants.

### Identificcation of DEGs

The gene expression profiles GSE61592, containing microarrays of cardiac tissue samples from a mice model of MI/RI and normal mice cardiac tissue samples, were obtained from the Gene Expression Omnibus (GEO) database, using GPL96 platform for acquiring profile data. The data was then analyzed using R software (version 3.26.9) and the limma package, expressed as the volcano and heat maps. It was followed by applying log transformation DEGs in the profile and the student's *t*-test analysis. The expression data of the profile was differentially analyzed with |log2 Fold Change (FC)| > 2.0 and p < 0.05 as thresholds to screen DEGs.

### Pathway analysis

Kyoto Encyclopedia of Genes and Genomes (KEGG) for DEGs was performed via an online tool DAVID (https://david.ncifcrf.gov/), which was used to identify molecular interaction and relation networks. The significantly different signal pathways were screened by the threshold p<0.05, and the top significantly enriched analysis results were displayed.

### Construction of protein-protein interaction (PPI) network

The PPI network diagram of cuproptosis-related genes was constructed via STRING online analysis network (http://www.string-db.org/), and the results were analyzed using Cytoscape 3.9.0.

### Correlation analysis between cuproptosis-related genes

The association of the cuproptosis-related genes was analyzed using Spearman's rank correlation analysis in R software, which was visualized using the “ggplot2” package.

### MI/RI mice model

After one week of acclimatization, mice were randomly divided into control and model groups (n = 6). To establish the I/R model, animals were anesthetized by intraperitoneal injection of 1% sodium pentobarbital (75 mg/kg) and then connected to a ventilator (respiratory rate 115, respiratory ratio 1.3:1, tidal volume 2.0). The heart was exposed by thoracotomy between the 3^rd^ and 4^th^ ribs of the mouse, the open-heart capsule was torn, and the left anterior descending branch was ligated with 8‒0 needle suture. ST-segment elevation was confirmed as ischemia by ECG. After 45 min ischemia, the ligation was removed, the rib space was closed, and the muscle and skin were sutured for 24h reperfusion, followed by collecting myocardial tissues for PCR analysis.

### RT-qPCR

The myocardial tissues of mice or serum samples of patients were mixed with TRIzol® reagent (Beyotime, Shanghai, China) to extract the total RNA. Then, a reverse transcription kit (Vazyme Biotech Co., Ltd, Nanjing, China) was used to perform the reserve transcription to obtain the cDNA. Primers were synthesized by Genscript Biotech (Nanjing, China). The mRNA expression of related genes was performed by qRT-PCR with a 20 μL reaction system containing specific primers and SYBR Green Master Mix (Vazyme) using CFX96 Real-Time PCR Detection System (Bio-Rad, USA). GAPDH was used as an internal reference. The indicated genes' Cycle Threshold (CT) values were calculated using the 2^-ΔΔCt^ method. Primer sequences are as follows (5′→3′).

Pyruvate dehydrogenase B (Pdhb): forward AAGAGGCGCTTTCACTGGAC, reverse ACTAACCTTGTATGCCCCATCA.

Dihydrolipoamide S-acetyltransferase (Dlat): forward CGGAACTCCACGAGTGACC, reverse CCCCGCCATACCCTGTAGT.

Pyruvate dehydrogenase E1 subunit alpha 1 (Pdha1): forward TGGTAGCATCCCGTAATTTTGC, reverse ATTCGGCGTACAGTCTGCATC.

GAPDH: forward TGTGGGCATCAATGGATTTGG, reverse ACACCATGTATTCCGGGTCAAT.

### Statistical analysis

Data were analyzed using GraphPad (version 6.0) and expressed as the mean ± Standard Deviation (SD). Differences were analyzed using a student's *t*-test. Receiver Operating Characteristics (ROC) curve analysis was conducted to predict the diagnostic value of Pdhb, Dlat, and Pdhα1. Differences were deemed as statistically significant at p<0.05.

## Results

### DEG levels in the myocardial tissue of the ischemia-reperfusion injury mice

**Fig. 1:**
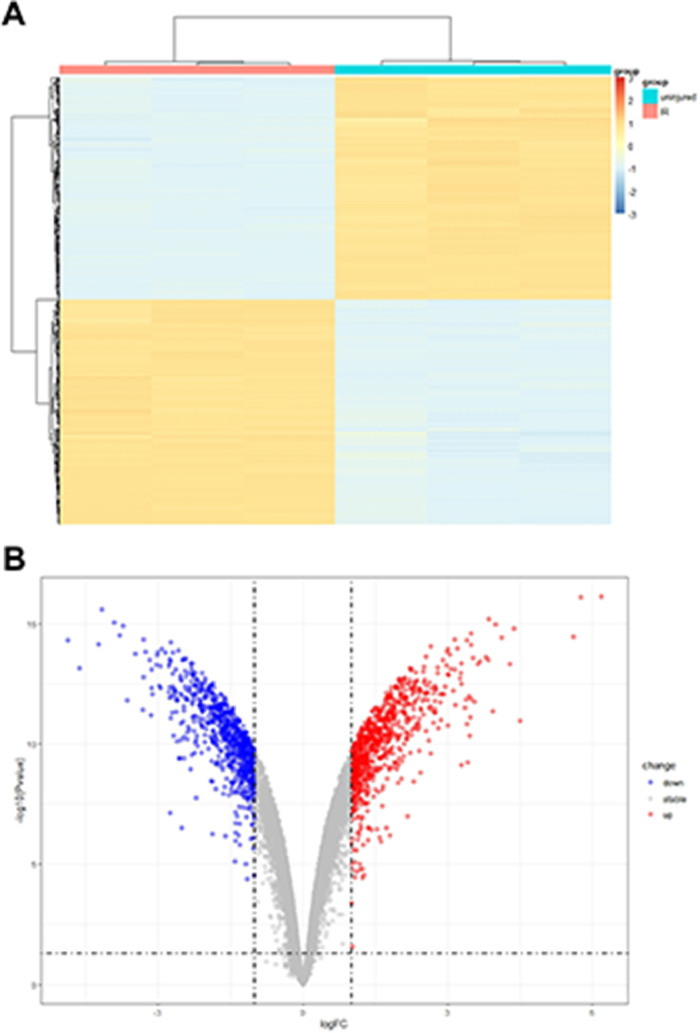
DEG levels in the myocardial tissue of the ischemia-reperfusion injury mice. Analyzing the microarray datasets, GSE61592 showed the differently expressed genes in the myocardial tissue of the ischemia-reperfusion injury mice as heat map (A) and volcano map (B).

**Fig. 2:**
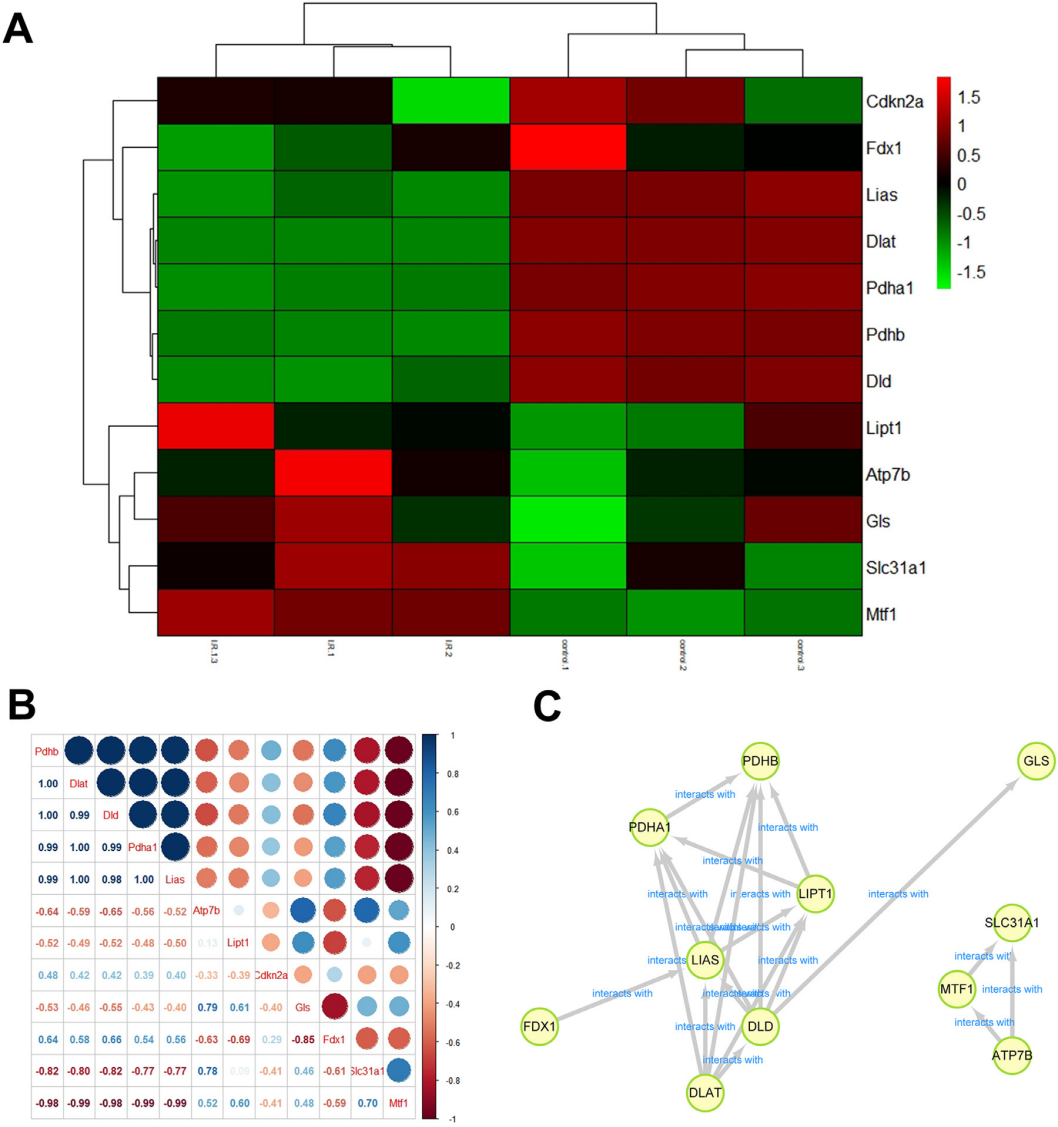
Expressions of cuproptosis related genes. (A) The cuproptosis-related gene expressions in the MI/RI are shown as heat maps. (B) The association of the cuproptosis related genes. (C) The PPI network of the cuproptosis related genes.

The analysis of microarray datasets GSE61592 revealed a total of 798 upregulated and 768 downregulated genes in the myocardial tissue of the ischemia-reperfusion injury mice, which was shown as the heat map (Fig. 1A) and volcano map (Fig. 1B). The cuproptosis related gene expressions in the MI/RI were also analyzed using the microarray datasets GSE61592, shown as the heat map in Fig. 2A. Cdkn2a, Fdx1, Lias, Dlat, Pdhb and Dld were decreased. ischemia-reperfusion injury mice, while Lipt1, Atp7b, Gls, Slc31a1 and Mtf1 were significantly increased. The association of the cuproptosis-related genes is shown in Fig. 2B; blue indicates a positive correlation, brown indicates a negative correlation, and the interaction between cuproptosis-related genes is shown in Fig. 2C. These results suggested that the cuproptosis-related genes were closely related to the MI/RI.

### Dlat, Pdhb, and Pdhα participated in the MI/RI

**Fig. 3:**
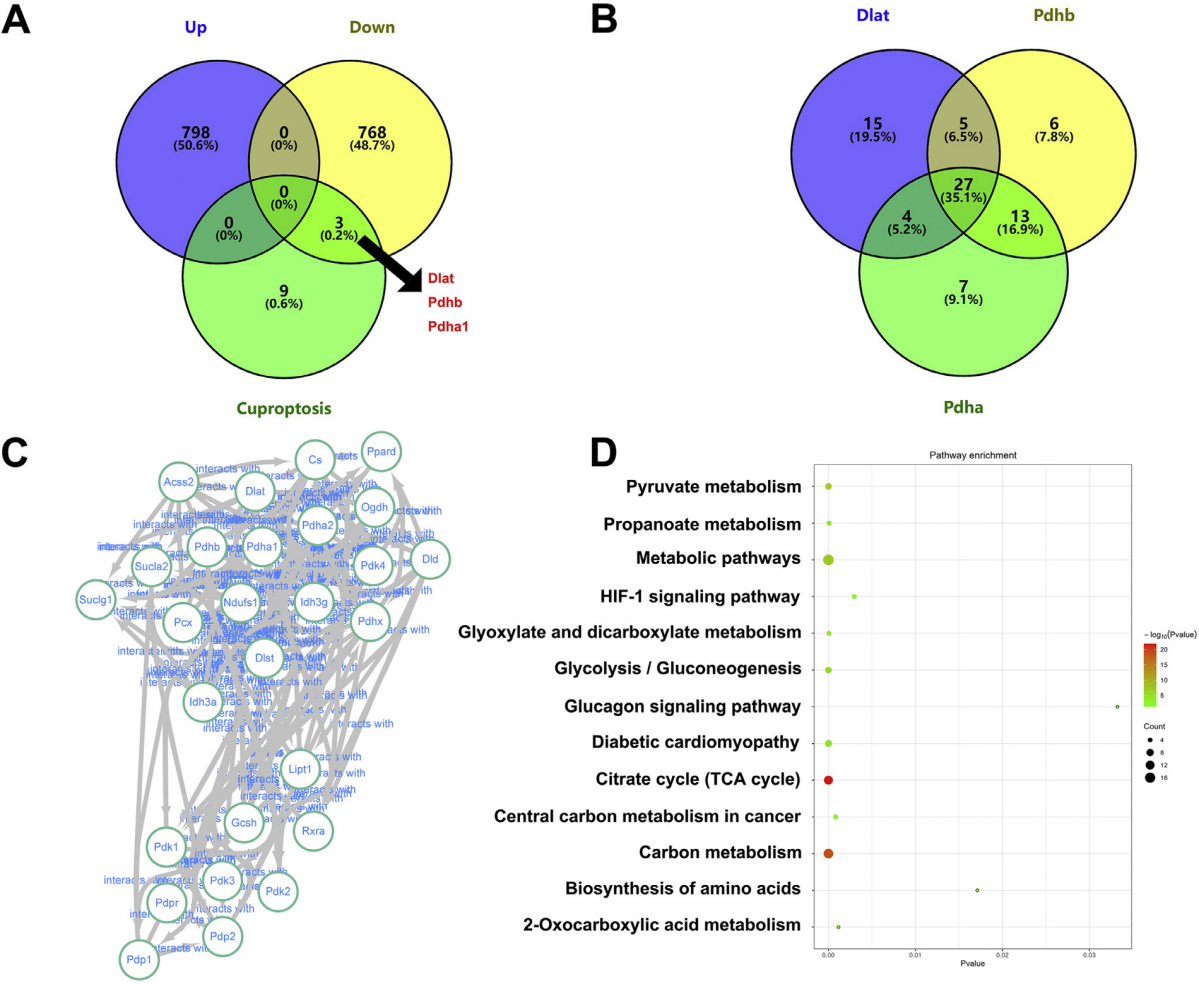
Dlat, Pdhb, and Pdhα participated in the MI/RI. (A) The Venn Diagram of the differently expressed genes in MI/RI and cuproptosis related genes. (B) The Venn Diagram of the Dlat, Pdhb, and Pdhα-related top 50 proteins. (C) The PPI network of the 27 proteins commonly associated with Dlat, Pdhb, and Pdhα. (D) KEGG analysis of the 27 proteins commonly associated with Dlat, Pdhb, and Pdhα.

The analysis of the function of cuproptosis-related genes in the MI/RI, through the Venn diagram, revealed that cuproptosis-related genes (Dlat, Pdhb, and Pdhα) belonged to the 768 down-regulated genes in the myocardial tissue of the ischemia-reperfusion injury mice (Fig. 3A). Moreover, the Dlat, Pdhb, and Pdhα-related top 50 proteins were expressed, among which 27 proteins were found on which Dlat, Pdhb, and Pdhα acted together (Fig. 3B). The PPI network of 27 proteins commonly associated with Dlat, Pdhb, and Pdhα is shown in Fig. 3C. Additionally, the KEGG analysis demonstrated that genes regulated by Dlat, Pdhb and Pdhα were primarily enriched in pyruvate metabolism, propanoate metabolism, metabolic pathways, HIF-1 signaling pathway, glyoxylate and dicarboxylate metabolism, glycolysis/gluconeogenesis, glucagon signaling pathway, citrate cycle (TCA cycle), central carbon metabolism in cancer, carbon metabolism, biosynthesis of amino acids, and 2-oxocarboxylic acid metabolism (Fig. 3D).

### Dlat, Pdhb, and Pdhα1 might serve as prognosis and diagnosis biomarkers of MI/RI

**Fig. 4:**
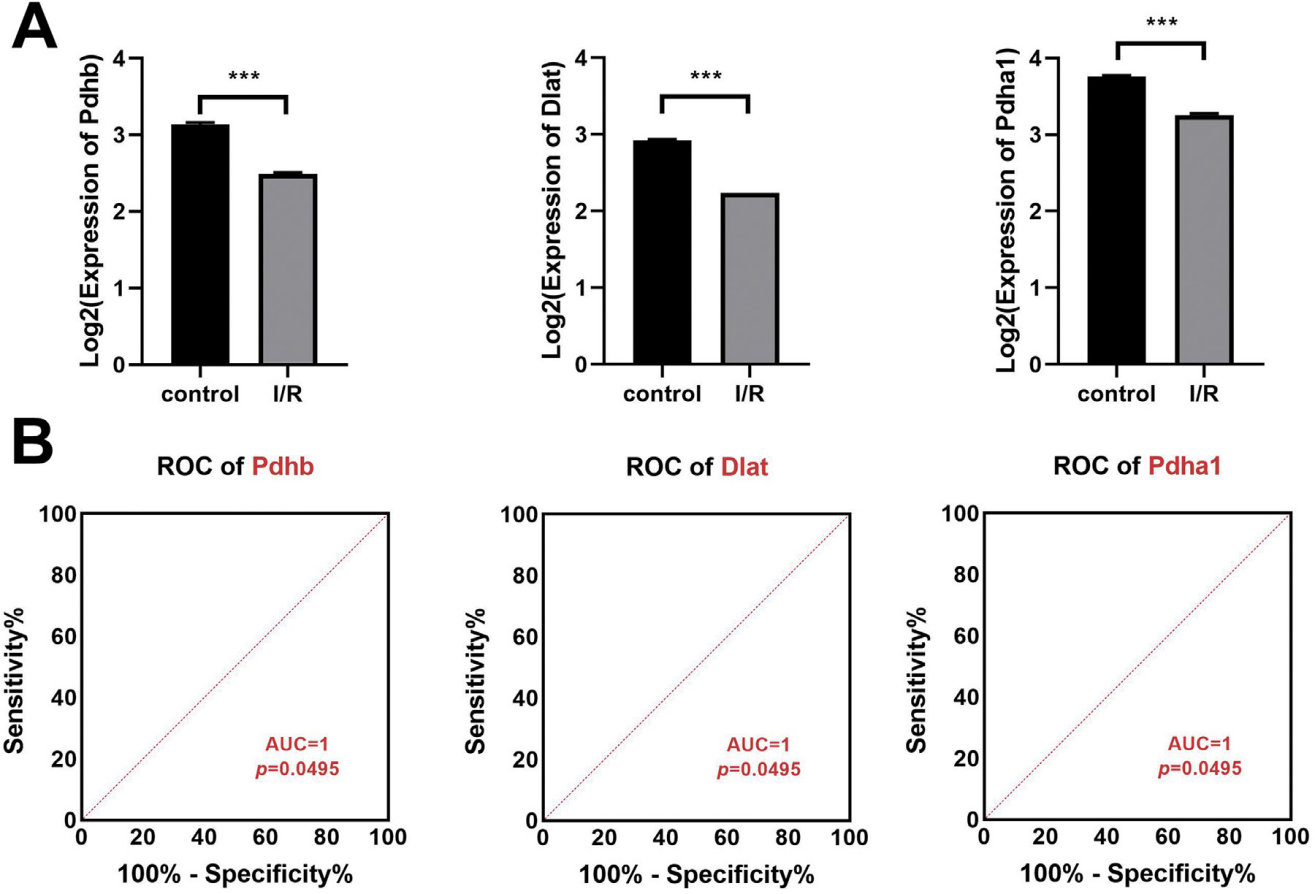
Dlat, Pdhb, and Pdhα1 might serve as prognosis and diagnosis biomarkers of MI/RI. Analyzing the microarray datasets GSE61592, (A) the Dlat, Pdhb, and Pdhα1 expressions in the myocardial tissue of the ischemia-reperfusion injury mice was obtained. (B) The AUC of Dlat, Pdhb, and Pdhα1 in myocardial tissue of the ischemia-reperfusion injury mice was shown (*** p < 0.001).

The Dlat, Pdhb, and Pdhα1 expressions in myocardial tissue of the ischemia-reperfusion injury mice were obtained from microarray datasets GSE61592 and Dlat, Pdhb, and Pdhα1 expressions were significantly decreased in ischemia-reperfusion injury (p < 0.0001, Fig. 4A), where the AUC of Dlat, Pdhb, and Pdhα1 were found equal to 1 (p = 0.0495), as per Dlat, Pdhb, and Pdhα expressions (Fig. 4B).

### Pdhb was demonstrated to be a diagnosis biomarker of MI/RI

**Fig. 5:**
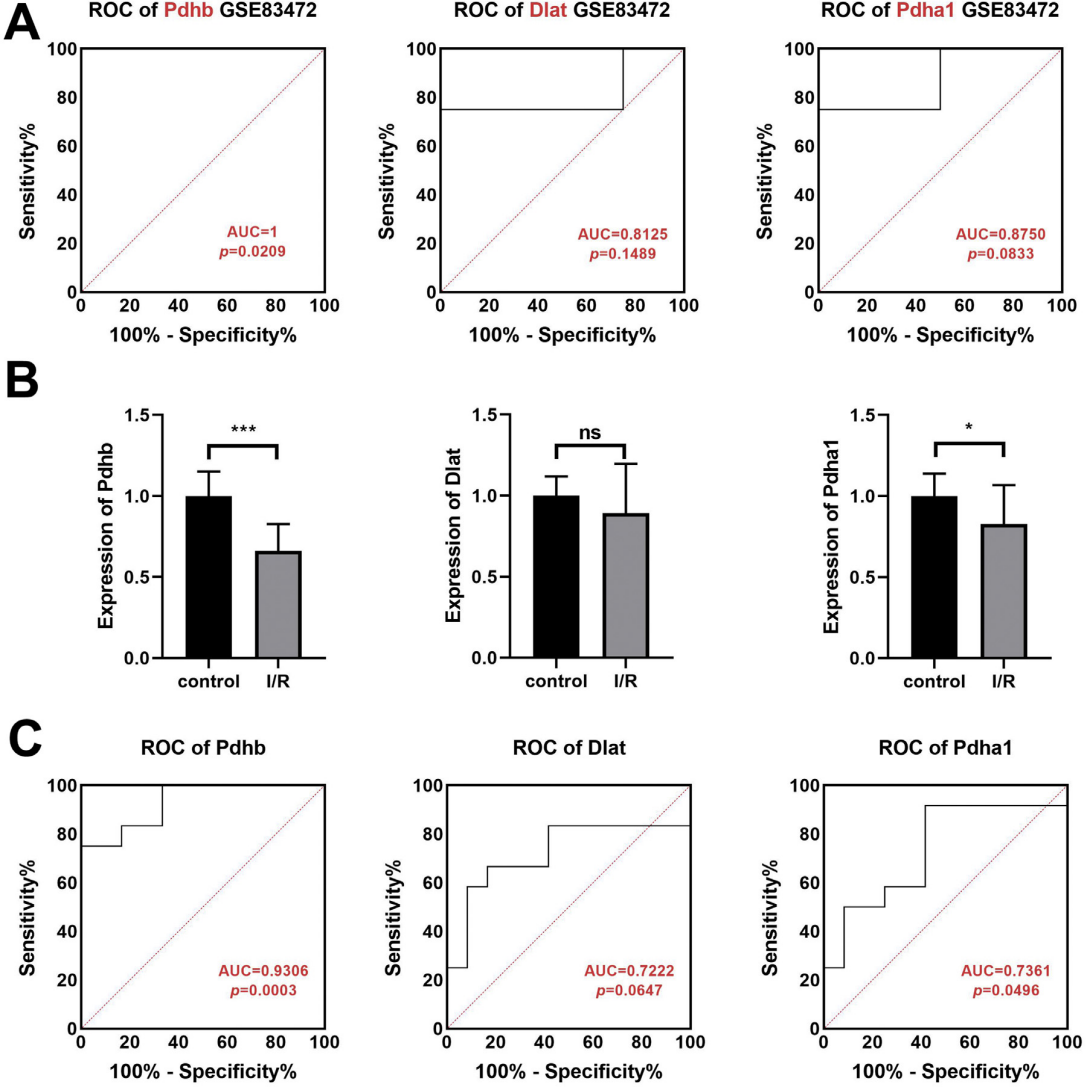
Pdhb was demonstrated to be a diagnosis biomarker of MI/RI. (A) Through analyzing the microarray datasets GSE83472, the Dlat, Pdhb, and Pdhα1 expressions in the myocardial tissue of the ischemia-reperfusion injury mice were obtained. (B) The Dlat, Pdhb, and Pdhα1 expressions in the myocardial tissue of the ischemia-reperfusion injury mice were detected by qPCR. (C) Receiver operating characteristics curve analysis was conducted to predict the diagnostic value of Pdhb, Dlat, and Pdhα1 (* p < 0.05, *** p < 0.001).

The sensitivity and specificity of Dlat, Pdhb, and Pdhα1 for diagnosing MI/RI using the microarray datasets GSE83472 were analyzed. Results showed the Pdhb AUC of 1 (p = 0.0209), while the Dlat and Pdhα1 AUC were found to be 0.8125 (p = 0.1489) and 0.8750 (p = 0.0833), respectively (Fig. 5A). Analyzing the expression levels of Dlat, Pdhb, and Pdhα1 in myocardial tissues of mice revealed dramatically decreased expression levels of Pdhb (p < 0.001) and Pdhα1 (p < 0.05), while Dlat showed no difference (p > 0.05) (Fig. 5B). Similarly, analysis of the sensitivity and specificity of Dlat, Pdhb, and Pdhα1 revealed a Pdhb AUC of 0.9306 (p = 0.0003), while the AUC of Dlat and Pdhα1 were found to be 0.7222 (p = 0.0647) and 0.7361 (p = 0.0496), respectively (Fig. 5A).

**Fig. 6:**
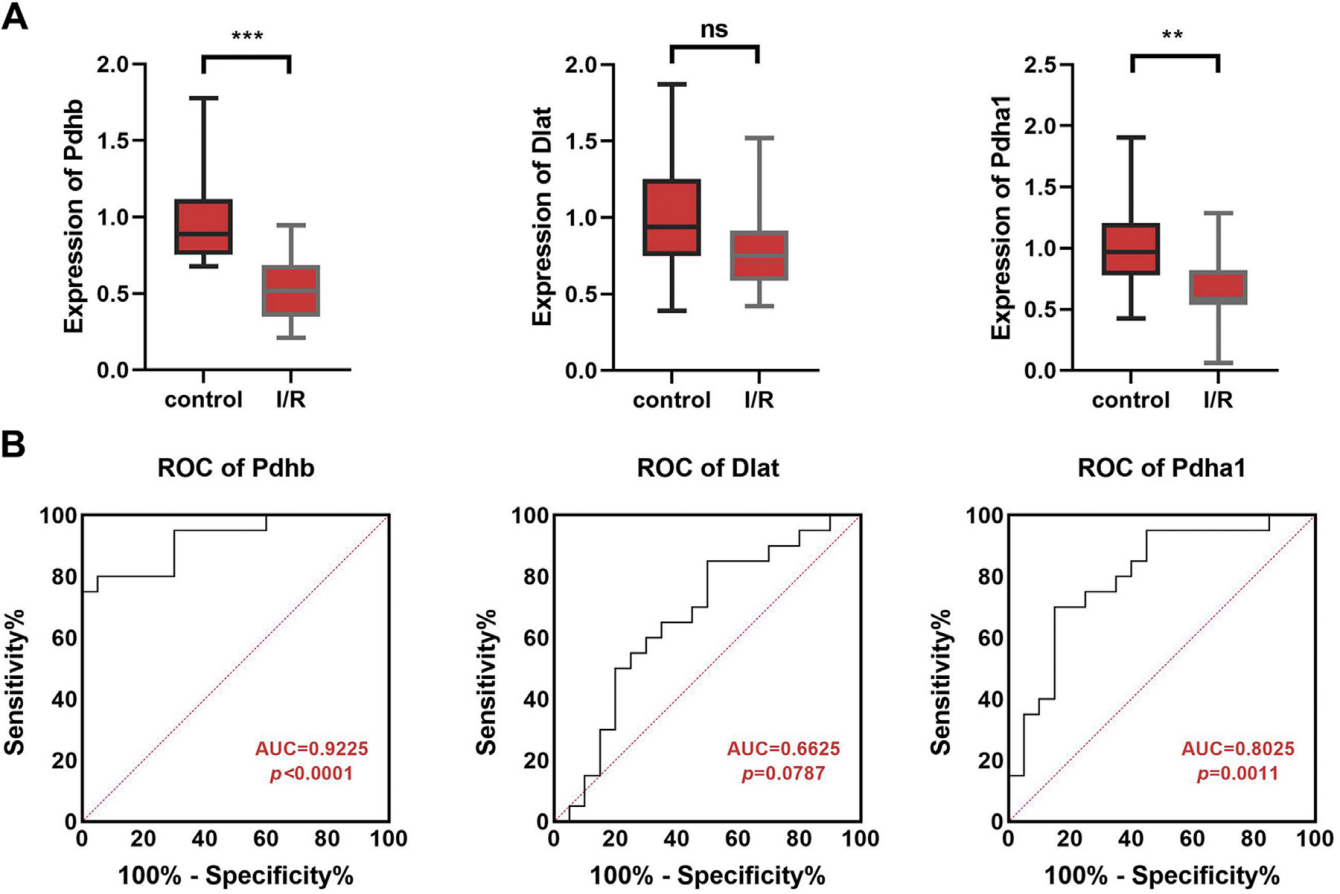
The expression and AUC value of Dlat, Pdhb, and Pdhα1 in MI/RI patients. (A) The Dlat, Pdhb, and Pdhα1 expressions in the serum samples of the ischemia-reperfusion injury patients were detected by qPCR. (B) Receiver operating characteristics curve analysis was conducted to predict the diagnostic value of Pdhb, Dlat, and Pdhα1 (** p < 0.01, *** p < 0.001).

In addition, the authors also found that Pdhb (p < 0.001) and Pdhα1 (p < 0.01) levels were significantly decreased in the serum of MI/IR patients and Dlat showed no difference (p > 0.05) (Fig. 6A). The ROC analysis of Dlat, Pdhb, and Pdhα1 levels in MI/RI patients showed the AUC of Pdhb was 0.9225 (p < 0.0001), Dlat was 0.6625 (p = 0.0787) and Pdhα1 was 0.8025 (p = 0.0011), respectively (Fig. 6B). These results indicated that Pdhb might be a diagnosis biomarker of MI/RI.

## Discussion

In this study, the gene expression profiles of the myocardial tissue in the ischemia-reperfusion injury mice from GEO datasets GSE61592 were analyzed. Results showed a total number of 798 upregulated and 768 downregulated genes, where Dlat, Pdhb, and Pdhα1, and cuproptosis-related genes were found to belong to the downregulated genes in MI/RI, where Pdhb demonstrated to be a diagnosis biomarker of MI/RI.

The content of copper in the body remains relatively stable. The decrease in copper content destroys the function of critical metal-binding enzymes, while the excessive increase results in abnormal cell function and death.^18^ The body's intake, excretion, and metabolism of copper are regulated and maintained by various factors. When the copper homeostasis in the body is broken, abnormal copper metabolism or copper-induced cell death will lead to a series of diseases.^17^^,^^19^ For example, Bian et al.^20^ demonstrated that the cuproptosis-related gene functioned as a potential prognostic predictor for clear cell renal cell carcinoma, providing novel insight for precise treatment. Similarly, Lv et al.^21^ confirmed that LIPT1, one of the cuproptosis-related genes, exhibited a prognostic value in skin cutaneous melanoma and demonstrated that LIPT1 was closely related to the immune infiltration of the skin cutaneous melanoma. However, the role of cuproptosis in MI/RI has not been reported. Here, it was observed that Dlat, Pdhb, and Pdhα1, cuproptosis-related genes were dramatically decreased in the MI/RI through the datasets GSE61592 analysis, preliminarily indicating that Dlat, Pdhb, and Pdhα1 might drive the MI/RI occurrence.

Pdhb is the key rate-limiting enzyme that decarboxylates glucose-derived pyruvate to form acetyl-CoA during the oxidative phosphorylation process of the body.^22^ Pdhα1 is a carrier gene encoding an essential subunit of pyruvate dehydrogenase-1, a hub connecting glycolysis and TCA cycle, and a key gene for energy regulation.^23^ Studies have shown that Pdhα1 gene deficiency can cause Leigh syndrome, lactate accumulation in the neuromuscular system, tumorigenesis, etc.^24^^,^^25^ Dlat belongs to the E2 subunit of the pyruvate dehydrogenase complex, which plays a crucial catalytic role in the conversion of pyruvate to acetyl CoA and is the only way for pyruvate to be converted to acetyl CoA after entering mitochondria.^26^ The enzyme encoded by Dlat determines whether the glucose-derived energy supply material can smoothly enter the citric acid cycle oxidative phosphorylation pathway for complete hydrolysis to generate energy or provide more synthetic lipids for cells.^27^ Genome-wide CRISPR-Cas9 function loss screening identified specific metabolic pathways mediating cuproptosis. The researchers treated human ovarian cancer cells with two copper ionophores, alismo and DTC, respectively, and identified ten genes that may be related to cuproptosis, including seven positive regulatory and three negatively regulated genes, where Dlat, Pdhb, and Pdhα1 belong to the positive regulatory genes.^28^ The present results indicated that Dlat, Pdhb, and Pdhα1 showed high sensitivity and specificity in MI/RI through the GSE61592 datasets. Besides, through the GSE8347 datasets, Pdhb was found to show high sensitivity and specificity.

To further verify the diagnostic value of Dlat, Pdhb, and Pdhα1 in MI/RI, an MI/RI mice model was established, and results showed that Pdhb and Pdhα1 were dramatically decreased. In contrast, Dlat showed no difference in the mice myocardial tissue, with significant sensitivity and specificity observed for Pdhb. These results indicated that Pdhb might be a diagnosis biomarker of MI/RI.

However, there are still some limitations in this study. The authors need to collect more clinical samples to analyze diagnostic value and conduct more profound mechanism research to clarify the specific mechanism of cuproptosis in MI/RI in future research.

In conclusion, this study demonstrated the prognostic value of cuproptosis-related genes (Dlat, Pdhb, and Pdhα1), especially Pdhb, which provided new insight into the MI/RI treatment.

## Authors' contributions

Rong Huang and Rongfeng Xu performed the bioinformatics analysis.

Rui Zhang and Wenjie Zuo contributed to the collection of clinical samples.

Zhenjun Ji and Zaixiao Tao contributed to the mouse model establishment.

Rong Huang and Yongjun Li performed the qPCR detection.

Genshan Ma designed the study and wrote the manuscript.

## Declaration of competing interest

The authors declare no conflicts of interest.
